# Control of *Penicillium glabrum* by Indigenous Antagonistic Yeast from Vineyards

**DOI:** 10.3390/foods9121864

**Published:** 2020-12-14

**Authors:** Catalina M. Cabañas, Alejandro Hernández, Ana Martínez, Paula Tejero, María Vázquez-Hernández, Alberto Martín, Santiago Ruiz-Moyano

**Affiliations:** 1 Nutrición y Bromatología, Escuela de Ingenierías Agrarias, Universidad de Extremadura, Avd. Adolfo Suárez s/n, 06007 Badajoz, Spain; ccabaasc@alumnos.unex.es (C.M.C.); amartinehi@alumnos.unex.es (A.M.); patejeroc@gmail.com (P.T.); m.vazquez.hern@gmail.com (M.V.-H.); amartin@unex.es (A.M.); srmsh@unex.es (S.R.-M.); 2 Instituto Universitario de Investigación en Recursos Agrarios (INURA), Avd. de la Investigación s/n, Universidad de Extremadura, 06006 Badajoz, Spain

**Keywords:** biocontrol, grapes, mechanism of action, volatile organic compounds, *Penicillium*

## Abstract

Biocontrol is one of the most promising alternatives to synthetic fungicides for food preservation. *Botrytis cinerea*, *Alternaria alternata,* and *Aspergillus* section *Nigri* are the most concerning pathogens for grape development. However, frequently, other species, such as *Penicillium glabrum* in this study, are predominant in spoiled bunches. In this work, 54 native yeasts from vineyards were screened by direct confrontation in potato dextrose agar plates as antagonists against *P. glabrum*. Isolates of *Pichia terricola*, *Aureobasidium pullulans*, and *Zygoascus meyerae* were selected for their antagonist activity in vitro, plus isolates of *Pichia kudriavzevii*, *Hormonema viticola,* and *Hanseniaspora uvarum* were used as negative controls. However, in vivo, confrontations in wounded grapes showed disagreement with direct confrontation in vitro. *P. terricola*, *P. kudriavzevii*, *H. viticola*, *Z. meyerae,* and *H. uvarum* significantly reduced the incidence of *P. glabrum* on grapes. Nevertheless, *P. terricola*, *H. viticola,* and *H. uvarum* themselves spoiled the wounded grapes. Inhibitions were associated with different mechanisms such as the production of volatile organic compounds (VOCs), lytic enzymes, biofilm formation, and competition for nutrients. The isolates of *P. kudriavzevii* L18 (a producer of antifungal VOCs which completely inhibited the incidence of *P. glabrum*) and *Z. meyerae* L29 (with pectinase, chitinase and β-glucanase activity and biofilm formation which reduced 70% of the incidence of *P. glabrum*) are proposed as suitable biocontrol agents against *P. glabrum*.

## 1. Introduction

A vineyard environment is a microbiologically complex ecosystem that harbors a wide variety of species of bacteria, yeast, and filamentous fungi [1]. Factors such as grape varieties, the stage of maturation, cultural practices, and climatic and geographical conditions define the diversity of the microbial population [2,3]. Among filamentous fungi, a relevant number of species have been described to spoil grapes or cause rotting of different parts of the plant. *Alternaria alternata*, *Botrytis cinerea*, and *Cladosporium cladosporioides*, together with *Aspergillus* spp. and *Penicillium* spp. are frequently isolated from different vineyards [2,4,5].

*Botrytis cinerea*, the causal agent of *Botrytis* bunch rot, is the most concerning pathogen in grapes [6,7]. This pathogen overwinters as mycelium, sclerotia and/or necrotic tissues, and in spring, with favorable weather conditions, a large number of conidia are produced, which are dispersed by wind or water [8]. *Botrytis* bunch rot has a large impact on the quality of grapes and wines. Chemical changes, which include reduction of total phenolic compounds of seeds and skin because fungal laccases are well described [9]. Moreover, it is implicated in the production of earthy “off” aromas in wine by geosmin and other volatile compounds synthesis [10]. In addition, the presence of mycotoxigenic species such as *Aspergillus niger*, *Aspergillus carbonarius* and *Aspergillus tubingiensis* [2,11], which are producers of ochratoxin A (OTA), are other main sanitary concern in grape production. Although there is controversy about the prevalence of toxigenic strains, a higher percentage of toxigenic strains for *A. carbonarius* than other Aspergilli has been recorded [12]. *Aspergillus* species growth and OTA production are conditioned by climatology. In this sense, wines from the Mediterranean area are prone to accumulate OTA because the mold development is favorable [13]. Moreover, *Aspergillus* infections have an impact on the chemical composition of grapes [14] and wines, increasing levels of acids and terpenes [15]. Among *Penicillium* species, *Penicillium expansum* is currently receiving a great deal of attention because of the detection of patulin and citrinin in grapes and wines [16]. In addition, it is implicated, alone or combined with *B. cinerea*, in the production of earthy “off” aromas in wine by the production of geosmin [10]. Less attention has been paid to other spoiling species such as *Penicillium glabrum*, which together with other *Penicillium* spp. are responsible for secondary infections. Its presence has been widely described in the vineyards and the postharvest stage [4,5,17,18]. Bau et al. [19] reported that *Penicillium citrinum* and *P. glabrum* were the most frequently *Penicillium* isolated of 2800 grape berries from vineyards of different locations of the Spanish Mediterranean coast. Likewise, *P. glabrum* appeared as one of the most prevalent species of *Penicillium* in Brazilian [20] and Portuguese [21] grapes. Besides secondary infections in grapes, *P. glabrum* presence has been related to earthy, moldy, and mushroom odors associated with geosmin, 2-methylisoborneol, 1-octen-3-one, and 1-octen-3-ol [22] in must and wines [5] and other products [23]. Other studies showed the potential of *P. glabrum* to be an agent causing rotting in pomegranates [24,25] and onion bulbs [26].

Nowadays, the application of chemical substances is the most widely used method to control pre and postharvest fungal disease. However, residues of fungicides on food commodities and their involvement in environmental and human toxicity generate social concern [27]. Moreover, the development of resistance to fungicides by strains of pathogenic molds is a serious threat for managing the crops. Concretely, resistant strains of pathogenic species related to vineyards as *B. cinerea* [28], *P. expansum* [29] and *Aspergillus* spp. [30] have widely been documented. In recent decades, efforts to replace chemical fungicides with environmentally friendly and less toxic treatments have multiplied [31]. Standing out among them is biological control. The characteristics of ideal biocontrol agents enumerated by Droby et al. [32], such as genetic stability, effectiveness against a wide range of pathogens, non-detrimental to human health, allow reducing the use of chemical compounds. The complex microbial population present in vineyards and berry grapes harbor multiple species with antagonistic potential [1]. *Botrytis cinerea* has been the main target for biocontrol studies. Among bacteria, *Bacillus subtilis*, *Pantoea agglomerans*, *Pantoea ananatis*, *Pseudomonas fluorescens*, and *Acinetobacter lwoffii* isolated from vineyard environments have proved to have remarkable antagonistic activity against *B. cinerea* [6,33,34]. A high diversity of yeast species isolated from vineyards have been described as antagonistic agents against *B. cinerea*. Wang et al. [7] reported effective control by *Aureobasidium pullulans*, *Metschnikowia chrysoperlae*, *Metschnikowia pulcherrima*, *Meyerozyma guilliermondii,* and *Saccharomyces cerevisiae*. Volatile organic compounds produced by *Starmarella bacilaris* [35] and the competition for nutrients by *A. pullulans* [36] and *Hanseniaspora uvarum* [6] are effective ways to control *B. cinerea*. Different studies about the biological control of species of *Aspergillus* section *Nigri* producers of OTA are presented in the literature. *Bacillus amyloliquefaciens*, *B. pumilus,* and *Lactobacillus plantarum* [37,38] among bacteria; yeast species as *A. pullulans*, *Kluyveromyces thermotolerans, M. pulcherrima, H. uvarum* [39,40,41,42]; and filamentous fungi as *Acremonium cephalosporium* [43] were efficient antagonists against *A. carbonarius*, and *A. nigri*; besides of producing a remarkable reduction of OTA accumulation on grapes. Works directed to control *Penicillium* development in grapes and vineyards by antagonistic agents are limited and mainly focused on *P. expansum* [44,45,46] and to a lesser extent to *Penicillium commune* [39], *Penicillium digitatum* [47] are other species of this genus. Nevertheless, the biological control of *P. glabrum* has not been still addressed.

In this work, screening for antagonistic yeast detection was performed against *P. glabrum* isolated from spoiled grape bunches. A preliminary mechanism of activity characterization was performed. Finally, the antagonistic capability was evaluated by confronting yeasts and molds in grapes.

## 2. Material and Methods

### 2.1. Sampling, Microbial Counts, and Isolation of Molds and Yeast

Samples were taken from one vineyard of the “Pardina” variety (white variety, *Vitis vinifera*). from Lobón (38°49′33.41′′ N; 6°37′34.78′′ W, Badajoz, Spain) in the year 2018. The maximum temperature observed in high summer in this region was 44.7 °C, and the annual accumulated precipitation was 488.6 mm (Köppen climate classification = Csa). Sampling was performed according to previous works [42]. Ten damaged and ten healthy grape bunches were selected at harvest time from twenty different plants across two diagonal transects. Samples were taken under aseptic conditions and were kept to <7 °C until analyses.

Microbial counts were performed by randomly selecting ten berry grapes from each bunch. After this, the samples were serially diluted with peptone water and plated in acidified potato dextrose agar (PDA, Condalab, Spain) to pH 3.5 with a sterilized solution of tartaric acid at 10% (*w*/*v*). The plates were incubated at 25 °C for 5 days, and the results were expressed as log_10_ CFU/g of grapes. The microbial analyses were performed in triplicate.

Isolation of yeasts and molds were performed based on colony morphology. Two-three colonies of each morphology and sample were selected from the highest diluted plates. Yeast isolates were selected from healthy bunches, whereas mold isolates were selected from damaged bunches. Each colony was streaked out on acidified PDA plates two consecutive times until pure colonies were obtained. After this, the isolates were stored in 40% sterile glycerol (*v*/*v*) at −80 °C.

### 2.2. Identification of Isolates

Genomic DNA from microbial isolates, yeast and molds, was extracted using the NucleoSpin^®^ Microbial DNA kit (Macherey–Nagel, Düren, Germany) according to the manufacturer’s instructions. To identify the isolates at the species level, the internal transcribed spacer ITS1/ITS2-5.8S rRNA region was amplified as detailed by Gallardo et al. [48] using the primer pairs ITS1 and ITS4 [49]. PCR reactions were run in a T100™ thermal cycler (Bio-Rad, Hercules, CA, USA) using an initial denaturation at 94 °C for 4 min, followed by 35 cycles of 94 °C for 1 min, annealing at 55 °C for 1 min, elongation at 72 °C for 1 min, and a final extension at 72 °C for 10 min. PCR products were purified using the GeneJET PCR purification kit (Thermo Fisher Scientific) and then sequenced at the Service of Bioscience Applied Techniques (STAB) of the University of Extremadura (Badajoz, Spain). The sequences were analyzed and edited with Chromas Pro version 1.49 beta (Technelysium, Queensland, Australia) and checked by nucleotide-nucleotide comparison with EMBL/GenBank databases using the BLAST algorithm. The identities of the isolates were determined based on the highest score. The accession numbers of the reference strains are included in the Results section.

Identities of mold isolates identified as *P. glabrum* were confirmed by amplification of the D1/D2 domain of the 26S large ribosomal subunit (LSU) of rRNA using the primers NL1 and NL4 following the PCR conditions described by Gallardo et al. [48], β-tubulin gene using primers Bet2a and Bet2b [50] and calmodulin gene using primers CMD5 and CMD6 [51]. PCR amplification was performed in 50 μL of reaction mixtures containing 10 ng of genomic DNA, 50 pmol of the forward primer and reverse primer, 0.2 mM of each dNTP, 0.1 vol of 10X PCR buffer, and 1.25 U Green DreamTaq DNA polymerase (Fermentas, Thermo Fisher Scientific Inc., MA, USA). PCR reactions and PCR amplicons were carried out and sequenced as above.

Additionally, strain typing into *P. glabrum* isolates was performed with inter single sequence repeat-PCR (ISSR-PCR) markers (CAG)_4_ (5′-ARRTYCAGCAGCAGCAG-3′), (GTG)_5_ (5′-GTGGTGGTGGTGGTG-3′), UBC809 (5′-AGAGAGAGAGAGAGAGG-3′), and UBC817 (5′-CACACACACACACACAA-3′) following the PCR conditions described by Gallardo et al. [48]. Amplification products were separated on a 2% agarose gel stained with Midori Green Advance (Nippon, Japan). The GeneRuler 100 bp plus DNA ladder (Thermo Fisher Scientific, San José, CA, USA) was used as the reference.

### 2.3. Screening for Antagonistic Activity by Direct Confrontation

*Penicillium glabrum* isolates M1307 and M204 were used as a target for the screening of the antagonistic activity of isolated yeasts.

Inocula of mold and yeast were prepared as follows: conidia suspensions were obtained by growing *P. glabrum* isolates on acidified PDA at 25 °C for 7 days. The conidia were collected in distilled water with 0.05% (*v*/*v*) Tween-80 (Scharlab, Spain). Finally, spores were counted in a Neubauer chamber and adjusted to 10^5^ conidia/mL.

The isolates of yeasts were grown in acidified PDA plates at 25 °C for 48 h. A loop of cells was collected and resuspended in 500 μL of sterile distilled water. Finally, the cells were counted in a Neubauer chamber and adjusted to 2 × 10^7^ cells/mL.

Determining the antagonistic capability of yeast strains against *P. glabrum* isolates was done by challenging the yeasts with pathogenic mold isolates on acidified PDA plates. Two hundred microliters from a conidia solution were added to the surface of each agar plate and spread out. After drying, 5 μL of yeast suspension from each isolate were placed on agar plates. As controls, agar plates seeded with conidia solutions from each pathogenic mold isolate were inoculated with 5 μL of sterile distilled water instead of the yeast suspension. The plates were incubated for 5 days at 25 °C. Modifications in the growth of molds on the yeast plates were compared to the control plates. The confrontations were performed in triplicate. The yeast isolates that induced the largest modifications in mycelial growth were selected for subsequent assays. In addition, yeast isolates without remarkable interactions with *P. glabrum* were also selected as negative controls.

### 2.4. Characterization of the Mechanism of Antagonism

#### 2.4.1. Production of Antifungal Volatile Organic Compounds

A double-dishes system (DDS) of acidified PDA was used to determine the production of antifungal VOCs by yeast as described by Ruiz-Moyano et al. [52].

The diameter of the mycelia was measured daily. As a control, a DDS was performed without the inoculation of yeasts. The confrontations with all isolates of yeast were done in triplicate, and the trial was carried out twice. The lag phase (expressed in days) and growth rates (expressed as mm day^−1^) and the reduction of *P. glabrum* growth were calculated on the fourth day of confrontation. After this, the results were expressed as differences between confrontations and the control (without yeasts).

Analyses and identification of volatile compounds produced by yeasts were performed as Ruiz-Moyano et al. [52]. The extraction of compounds from DDS was conducted by solid-phase microextraction (SPME) with a 10 mm-long, 75 μm-thick fiber coated with Carboxen/polydimethylsiloxane (Supelco, Bellefonte, PA, USA). The analysis was done in a GC/MS using an Agilent 6890 GC-5973 MS system (Agilent Technologies, Little Falls, DE, USA) equipped with a 5% phenyl-95% polydimethylsiloxane column (30 m × 0.32 mm inner diameter, 1.05 μm film thickness, Hewlett-Packard, Palo Alto, CA, USA).

#### 2.4.2. Effect of Antagonistic Yeasts on Spore Germination of Pathogenic Molds

Effect of yeast on conidia was performed according to Ruiz-Moyano et al. [53]. Aliquots (100 μL) of a suspension of 10^5^ conidia/mL of *P. glabrum* isolates were spread out on acidified PDA plates. After drying, 100 mL of selected yeast solutions (2 × 10^5^, 2 × 10^6^, 2 × 10^7^ cells/mL) were spread out in the same plate. The plates were incubated at 25 °C. Over 100 conidia were observed to assess the germination rate by measuring the germ tube at 10 h. The reduction in the percentage of spore germination was calculated by comparison with the control plates without yeast inoculation. In addition, the size of the germ tubes was compared with controls without inoculation of yeasts. Germ tubes were measured by observation and analyses at 20X magnification with sub-stage illumination (DMLS, Leica, Buccinasco, MI, Italy) using a Leica DM 2000 LED microscope. The experiment was performed in triplicate.

#### 2.4.3. Parasitism of *P. glabrum* hyphae

The determination of the interaction between selected yeasts and *P. glabrum* isolates was evaluated on PDA plates (8 mL per plate) according to Ruiz-Moyano et al. [53]. Samples of 5 μL of conidia suspensions were inoculated in the middle of the plates. Then, 5 μL of yeast cell suspensions (10^6^ cells/mL) were inoculated at the border of the mold inocula. The interaction was observed after 12, 24, and 48 h of incubation at 25 °C. Then the cultures were washed under tap water for 60 s, and the plates were observed with a Leica DM 2000 LED microscope.

#### 2.4.4. Production of Antifungal Extracellular Substances

##### Antibiosis

The capacity to produce extracellular antifungal substances was evaluated by subculturing selected yeast in a liquid medium. Sterile yeast extract peptone dextrose broth (YPD, Condalab), potato dextrose broth (PD, Condalab), and yeast malt broth (YM: yeast extract, 3 g/L; malt extract, 3 g/L; peptone, 5 g/L; and glucose, 10 g/L; Condalab) were used for this assay. Three different inocula were performed in 10 mL of culture broth, incubated at 25 °C for 2 days: 50 μL of selected yeasts (2 × 10^7^ cells/mL), co-inoculation of selected yeasts and conidia (50 μL of 10^5^ conidia/mL), and inoculation of selected yeast in culture broth supplemented with inactivated hyphae. The inactivation on hyphae consisted of the cultivation of 50 μL of conidia solution on 10 mL of culture broth at 25 °C for 5 days at 100 rpm. After this, the cultures were autoclaved at 121 °C for 16 min. Then, 1 mL of broth was centrifuged to 5000× *g* for 5 min. The pellet was used to supplement the 10 mL of culture broth.

After incubation, 5 mL were centrifuged at 10,621× *g* for 5 min, and supernatants were sterilized by filtration (22 μM of pore size). Sterile paper discs were immersed in the extracellular extracts for 5 s. After this, the discs were set in PDA plates previously seeded with 100 μL of 10^5^ conidia/mL. Plates were incubated at 25 °C for 48 h, and alterations in mycelia growth and inhibition halos were recorded.

##### Production of Lytic Enzymes

Three different enzymatic activities related to the degradation of cell walls were tested. Five μL of cell solution (10^7^ cells/mL) were inoculated in each culture medium. Chitinase activity and β-glucanase activity were determined, as previously described by Cordero-Bueso et al. [54], using YPD agar (Condalab) supplemented with 0.2% of chitin (Sigma, St. Louis, MO, USA) and 0.2% β-glucan (Sigma, St. Louis, MO, USA), respectively and revealed with Congo red. The presence of red halos was recorded. Pectinase activity was analyzed in plates of YPD agar supplemented with 10% apple pectin (Sigma, St. Louis, MO, USA). After 5 days of incubation at 25 °C, plates were flooded with 10% cetyl trimethyl ammonium bromide (CTAB). The presence of clear halos was recorded. Finally, protease activity was evaluated in 10% skim milk powder (AppliChem, Darmstad, Germany) and 2% of bacteriological agar [55]. The assays were repeated three times.

#### 2.4.5. In Vitro Biofilm Formation

The determination of in vitro biofilm formation was performed as detailed by Cordero-Bueso et al. [54]. Briefly, 10 μL of fresh suspension were inoculated in 1 mL of yeast nitrogen base (YNB, Condalab) with 100 mM glucose and incubated overnight at 28 °C. Subsequently, the tubes were centrifuged at 2935× *g* for 5 min. The supernatant was discarded, and the pellets were washed twice with a 1X phosphate-buffered saline solution (PBS, Thermo Fisher Scientific, Madrid, Spain) and resuspended in YNB + glucose (100 mM) medium to obtain 10^7^ CFU/mL.

The adhesion phase was achieved by inoculating 100 μL of the cell suspension into a 96-well polystyrene plate with a flat bottom (Thermo Fisher Scientific) at 28 °C and shaking in a shaker at 90 rpm for 3 h. The wells were washed twice with 150 μL of PBS, then 100 μL of the same medium were added into each well, and the plate was incubated at 28 °C in a shaker at 90 rpm for 72 h. The medium was taken daily and replaced by 100 μL of fresh YNB + glucose. After incubation, the wells were washed twice with 150 μL of PBS; then 100 μL of crystal violet 0.4% (*w*/*v*) were put into each well for 45 min. Not-fixed colorant was washed four times, with 150 μL of sterile distilled water, and, finally, 200 μL of 95% (*v*/*v*) ethanol was added. After 45 min, 100 μL of solution were transferred to a new polystyrene 96-well plate and measured at 590 nm. The absorbance values were subtracted from the control test values without yeast inoculation. Each yeast strain was evaluated in triplicate.

#### 2.4.6. Competition for Nutrients

Two methods were performed for the evaluation of competition for nutrients. First, depletion of iron was evaluated as previously described by Cordero-Bueso et al. [54] using PDA plates and PDA supplemented with 5 and 20 μg/mL of FeCl_3_. One hundred mL of spore suspension (10^5^ conidia/mL) were spread on plates. Then, 5 μL of yeast cell solutions (10^7^ cells/mL) were inoculated on PDA plates over spore suspensions. The plates were incubated at 25 °C for 7 days; then, a comparison of the inhibition halos was recorded.

The second method for the antagonistic mechanism based on competition for nutrients was an inoculation assay on wounded grapes performed as previously described by Ruiz-Moyano et al. [53]. Grapes were sterilized by immersion for 3 min in 100 ppm sodium hypochlorite, then rinsed with sterile distilled water and dried by air. One lesion was made in each berry with a sterilized tip (3 mm-wide × 3 mm-deep). Treatments involved the inoculation of 10 μL of 2 × 10^7^ cells/mL of selected yeasts, followed by the addition of 3 μL of 10^5^ conidia/mL of molds. The confrontations were also repeated by adding 10 μL of yeast nitrogen base (Condalab) in independent treatments. As controls, molds were inoculated alone.

Each inoculated grape was set in a sterile plastic bottle at 25 °C for 5 days. Periodically, the percentage of infected lesions was recorded. A significant increment of incidence of *P. glabrum* by the addition of YNB was recorded as positive competition for nutrients. Ten grapes were used per treatment, and the assay was repeated three times.

#### 2.4.7. In Vivo Assay and Competition for Nutrients Determination

The in vivo assay consisted of a comparison of the incidence of each of the strains of *P. glabrum* in wounded grapes when the selected yeasts were confronted. The procedure was the same as described in the previous section without the addition of nutrients. Thirty wounded grapes were used in each assay, and the experiment was repeated 3 times.

#### 2.4.8. Analysis of Results

Statistical analysis of the data was performed with the SPSS software package for Windows version 19.0 (SPSS Inc., Chicago, IL, USA). Percentages were converted into Bliss angular values before analysis. Differences in the mean values of percentage of germination, germ tube size, lag phase, growth rate, inhibition of growth, and incidence of disease were tested by one-way analysis of variance and separated by Tukey’s honestly significant difference test (*p* ≤ 0.050).

## 3. Results

### 3.1. Microbial Counts and Species Identification of Yeasts and Molds

The mean counts of yeast were 6.66 ± 1.08 and 3.91 ± 2.73 log_10_ CFU/g (*p* = 0.021) in healthy and damaged bunches, respectively. No statistical differences were found in the mold population between healthy and damaged bunches, with values of 3.74 ± 2.57 and 4.39 ± 1.84 log_10_ CFU/g (*p* = 0.516), respectively.

A total of 54 yeasts were isolated from healthy grape samples. Molecular identification by sequencing of ITS1-5.8S rDNA-ITS4 gave six different species (Table 1). *Aureobasidium pullulans* and *P. terricola* (formerly *Issatchenkia terricola*) were isolated from all samples analyzed. This was followed by *Pichia kudriadzevii* (formerly *Issatchenkia orientalis*) isolated from 8 samples, *H. uvarum* (4 samples), *Hormonema viticola* (2 samples), and *Zygoascus Meyerae* (1 sample). All sequences presented identities above 99% with the accession number previously deposited on the GenBank database.

Regarding the mold population, a total of 33 isolates of molds were obtained from grape bunches with symptoms. These were clustered in four species (Table 1). *Penicillium glabrum* was the most isolated species; it was present in all damaged bunches. Four samples presented only morphologies compatible with *P. glabrum*. Moreover, *A. alternata* was detected in 4 damaged bunches; *Aspergillus nigricans* in two, and *Cladosporium cladosporioides* only in one damaged bunch.

**Figure 1:**
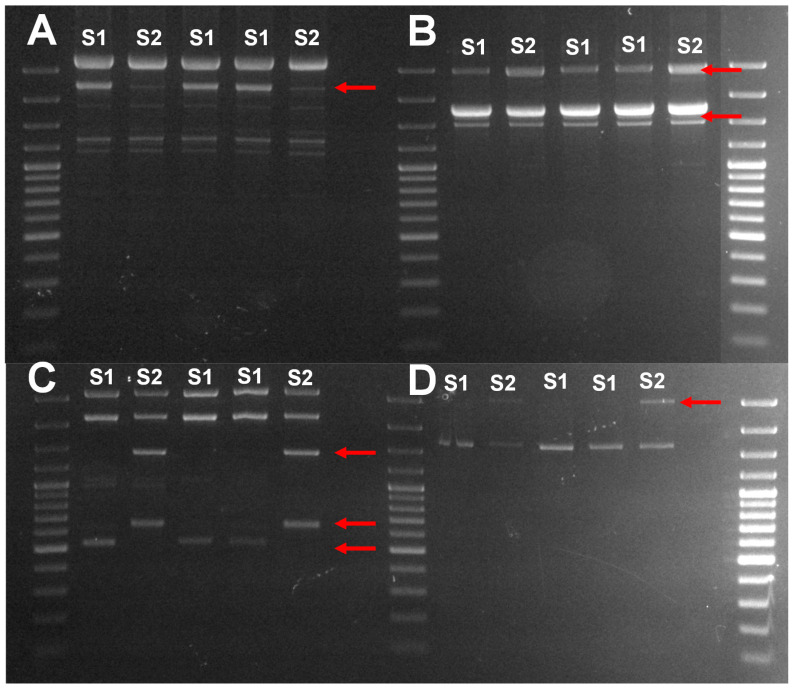
Agarose gel of ISSR-PCR profiles of 5 isolated of *P. glabrum* with primers (**A**) (CAG)_4_; (**B**) (GTG)_5_; (**C**) UBC809 and (**D**) UBC817. S1 and S2 track show profiles of the two genetic subgroups of *P. glabrum*.

According to the incidence of *P. glabrum* in damaged samples, this species was the objective for the biocontrol screening with the isolated yeasts. Accurate genetic identification of *P. glabrum* was performed in the 18 isolates by also sequencing large subunit (LSU) 26S rDNA, β-tubulin, and calmodulin genes. Identical sequences on ITS1-5.8S rDNA-ITS4 and LSU 26S rDNA were found in *P. glabrum* isolates. However, two different groups of sequences were found on β-tubulin (5 substitutions and one indel) and calmodulin (3 substitutions) sequences. Intraspecific differences were confirmed by comparison of profiles of four ISSR-PCR markers. The 18 isolates were grouped into the two intraspecific groups cited before based on the differences in one band with (CAG)_4_, two bands with (GTG)_5_, three bands with UBC809, and one band with UBC817 (Figure 1). One member of each genetic subgroup was selected for the screening of antagonistic activity with the selected yeasts.

### 3.2. Screening of Antagonistic Activity by Direct Confrontation

**Figure 2:**
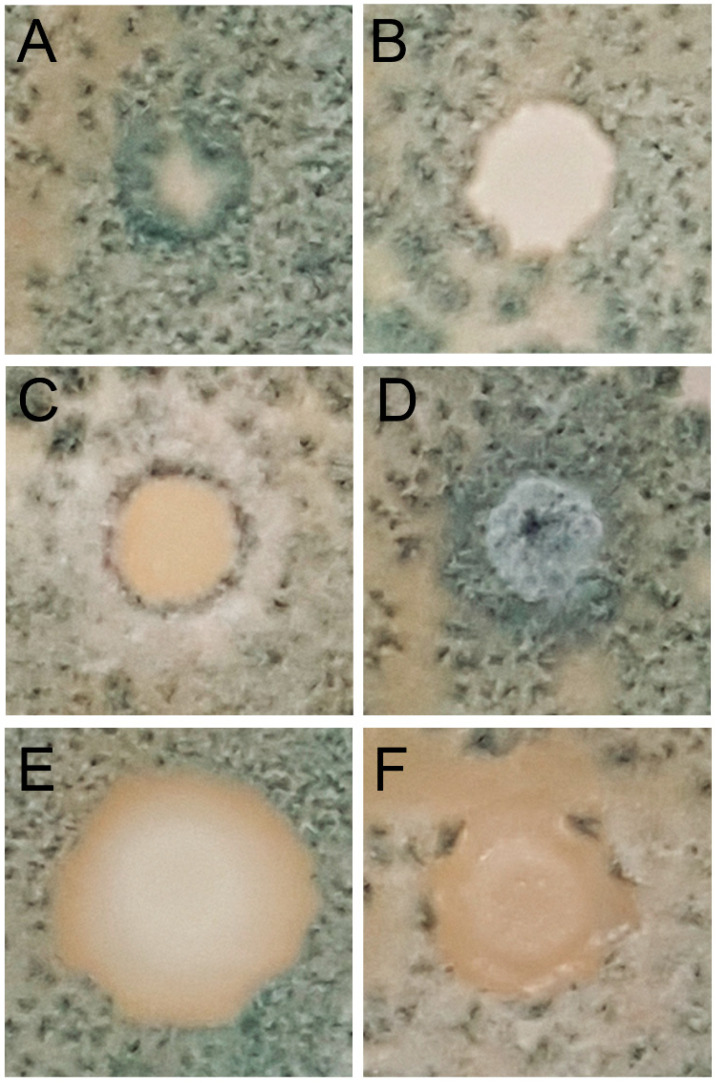
Images of direct confrontation between *P. glabrum* and *H. uvarum* (**A**), *H. viticola* (**B**), *A. pullulans* (**C**,**F**); *Z. meyerae* (**D**), and *P. terricola* (**E**).

The 54 isolates of yeast (Table 1) were confronted with the two *P. glabrum* strains selected. Different types of interactions were detected. Isolates belonging to *P. kudriavzevii* and *H. uvarum* species were covered by *P. glabrum* hyphae, as shown in Figure 2A. Other yeasts, such as *Z. meyerae* isolates, showed a neutral behavior (Figure 2B). These yeasts grew in the inoculation area and did not modify the mycelia. Most of *A. pullulans* isolates showed a null capacity for competition against *P. glabrum*, which completely covered areas of inoculation. However, two isolates showed a different interaction with the most intense sporulation appearing around the colony (Figure 2C) and inhibition halo (Figure 2F). *Hormonema viticola* isolates were covered by mold, although hyphae were visually modified (Figure 2D). Finally, seven out of 18 isolates of *P. terricola* presented a clear halo without hyphae surrounding the inoculation area. Visual appearance indicated that a clear zone could be associated with colonization of the space more than with the secretion of inhibitory substances. The isolates produced halo sizes ranging from 2 to 8 mm.

Based on results of direct confrontation with the two strains of *P. glabrum* resulting in the inhibition or modification of its growth, the isolates *P. terricola* L14, *A. pullulans* L30 and L31, and *H. viticola* L21 were selected for subsequent characterization of their antagonistic activity mechanisms. *P. kudriavzevii* L18, *Z. meyerae* L29, and *H. uvarum* L35 were used as negative controls for activity.

### 3.3. Characterization of the Mechanism of Antagonistic Activity against P. glabrum

First, regarding VOCs production by selected yeasts in control double dish systems without yeast, *P. glabrum* grew at 5.45 ± 0.07 (strain PG1307) and 5.51 ± 0.16 (strain PG204) mm day^−1^ at 25 °C. In the confrontations, the growth rate of *P. glabrum* 1307 significantly decreased with the volatile compounds produced by *P. kudriavzevii* L18 (*p* < 0.001) (Table 2). The rest of the confrontations did not inhibit mold growth rates; an increase in the growth rate was even detected for PG204. The lag phase of *P. glabrum*, 0.86 ± 0.01 day^−1^ for PG1307 and 0.61 ± 0.09 day^−1^ for PG204, were increased in the presence of volatile yeast compounds from *P. terricola* L14 and *P. kudriavzevii* L18. The values of the lag phase increased to 1.52 and 1.65 days^−1^ for PG1307 and to 1.02 and 1.35 days^−1^ for PG204, respectively. The lag phase of PG204 was significantly increased (*p* < 0.050) in the rest of the confrontations except in the presence of *Z. meyerae* L29.

Another parameter studied was the reduction of the mycelia diameter (Table 2). At 4 days of confrontation, *P. kudriavzevii* L18, through VOC production, caused the greatest inhibition of *P. glabrum* (31.48 ± 2.50% and 23.91 ± 1.68% for PG1307 and PG204, respectively). *Pichia terricola* L14 and *Z. meyerae* L21 also significantly reduced the diameter of both strains of molds. *Penicillium glabrum* 204 was inhibited by *A. pullulans* L31 and by *H. uvarum* L35 as well.

The inhibition of spore germination and a reduction in the size of the germ tube by selected yeasts is shown in Table 3. No differences were found in the percentage of spores germinated between both *P. glabrum* strains with 88.78 ± 4.03% for PG1307 and 93.74 ± 3.93% for PG204 after 10 h of incubation. The size of the germ tube was 71.61 ± 30.50 μM for PG1307 and 87.02 ± 42.20 μM for PG204. *Pichia terricola* L14 was the unique selected yeast that reduced the conidia germination. A significant reduction was obtained for the three concentrations of cells of *P. terricola* L14 assayed, and it was yeast concentration-dependent (*p* < 0.050). In addition, a reduction in the size of the germ tube was observed by confrontation with *P. terricola* L14 and *P. kudriavzevii* L18. Germ tubes in these confrontations had around half of the size of the controls. However, no significant influence of yeast cell concentration was observed on germination tube size.

The results for the other mechanisms of antagonism studied (parasitism, antibiosis, production of lytic enzymes, biofilm formation, and competition for nutrients) are shown in Table 4. Weak cell adhesion to *P. glabrum* hyphae was observed for *Z. meyerae* L29. The rest of the yeasts did not show the ability to attached to mold cell walls. Different enzymatic activities were observed. *Pichia terricola* L14 presented β-glucanase activity. *Hormonema viticola* L21, *Z. meyerae* L29, and A. pullulans L30 and L31 presented chitinase, β-glucanase, and pectinase activity. In addition, both strains of *A. pullulans* tested displayed proteolytic activity. The screening of biofilm formation in vitro assay showed that the strains *P. terricola* L14, *Z. meyerae* L29 and *H. uvarum* L35 were capable of adhering to the bottom of polystyrene wells and retain crystal violet colorant.

The iron competition was not the mechanism of any selected yeast in this study (Table 4). On grapes, the capacity for control of *P. terricola* L14 was reduced when YNB broth was added to the wound. All grapes presented mycelia development of *P. glabrum* after 3 days of storage when nutrients were artificially added, whereas around 10% of grapes presented symptoms of *P. glabrum* development without nutrient addition. Hence, competition for nutrients can be associated with this yeast strain. However, the rest of the yeasts confronted with *P. glabrum* in wounded grapes did not reduce (*p* < 0.050) their control capability when extra nutrients were present in the environment.

### 3.4. In Vivo Assay on Wounded Grapes

**Figure 3:**
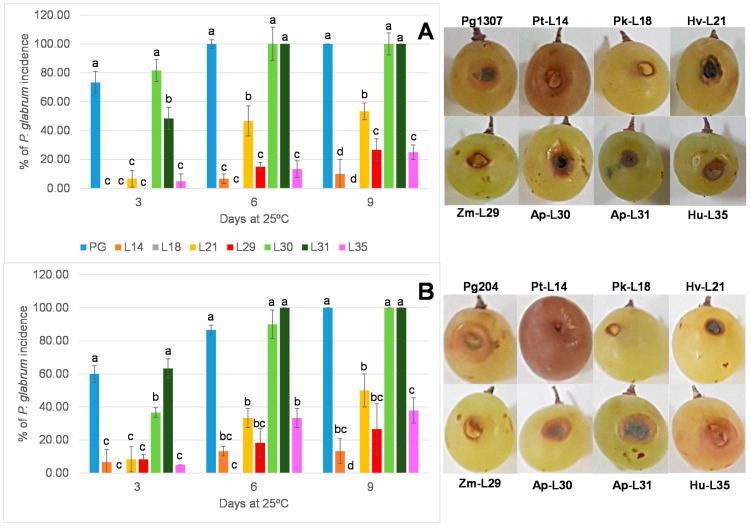
Percentage of disease incidence and symptoms of confrontations on wounded grapes at 25 °C among *P. glabrum* M1307 (**A**), M204 (**B**), and the yeast isolates *P. terricola* L14, *P. kudriavzevii* L18, *H. viticola* L21, *Z. meyerae* L29, *A. pullulans* L30 and L31, and *H. uvarum* L35. Different letters indicate statistical differences (*p* < 0.050) in each sampling day.

Control wounded grapes infected with *P. glabrum* strains PG137 and PG204 showed that the incidence of both strains was similar (PG1307, Figure 3A; PG204, Figure 3B). However, PG1307 was more infective (*p* < 0.050), achieving 100% of incidence after 6 days of inoculation, whereas this happened at 9 days with PG204. The confrontation of molds with the selected yeasts gave some surprising results. Yeasts without a priori antagonistic properties according to direct confrontation assay significantly reduced the incidence of *P. glabrum* on grapes and vice versa. Yeasts selected as neutral or “negative controls” such as *P. kudriavzevii* L18, *H. viticola* L21, and *H. uvarum* L35 reduced (*p* < 0.050) the incidence of *P. glabrum* to 0%, ~50%, and ~33%, respectively, at 9 days of confrontation. On the contrary, *A. pullulans* isolates did not reduce the incidence of molds, despite the fact that they were selected due to their control of mold growth in PDA plates. The other preselected antagonistic yeasts, *P. terricola* L14 and *Z. meyerae* L29 reduced the incidence of *P. glabrum* to ~13% and ~25%, respectively. Figure 3A,B reflect the observation of mycelia development on wounds. However, the visual appearance of grapes inoculated with selected yeast excluded several of them as potential biocontrol agents. *Pichia terricola* L14 completely turned grapes to a brown color, as the pictures in Figure 3 show. *Hormonema viticola*, a “black yeast” [56], produced blackening of the wounds. In addition, *H. uvarum* L35 produced browning and depression of the wounds. The control without inoculation of molds confirmed the spoiling capacity of these three yeast isolates.

## 4. Discussion

The yeast populations of grapes vary depending on practices, vectors, climate, geographical area, maturity state, etc. [1]. Although controversial, results and hypotheses about the influence of these factors in microbial populations are shown in the literature. Thus, great differences in yeast populations have been registered in previous works. Settanni et al. [57] reported high counts of yeasts in grapes from the Marsala wine area (Italy) with values that ranged from 3.54 to 6.92 log_10_ CFU/g, which agreed with the yeast population found in the present work. In contrast, lower counts (10^2^ to 10^4^ log_10_ CFU/g) of yeasts in healthy grapes from Texas (USA) were reported by Bougreau et al. [58]. Damaged grapes gave higher counts of yeasts than healthy grapes from Attica (Greece), according to Nisiotou and Nychas [59] and from Trento, Italy [60]; these are in contrast with the results obtained in the present work.

In this work, six different yeast species were identified; *A. pullulans* and *Pichia* spp. were predominant. Similar species biodiversity was detected by Cordero-Bueso et al. [54], who found seven, six, and five different species from conventional, organic, and biodynamical vineyards, respectively. In this case, *A. pullulans* and *H. uvarum* were the dominant species. In the same sense, Drumonde-Neves et al. [61] found that human activities increased yeast biodiversity. They described a total of 23 different species dominated by *H. uvarum* (66% of isolates) and *P. terricola* (11%) in different vineyards from Azores Archipelago, being the maximum diversity per vineyard of 11 yeast species. Other works have found more richness of species in vineyards with different farming systems [1], with between 9 and 17 different yeast species; however, that analysis included samples from different parts of the plant. Climate is another factor that defines yeasts biodiversity. Lobón (Badajoz, Spain) belongs to a climatic area with media temperatures in the summer around 30 °C and very scarce rainfall (471 mm). Rainfall levels could be associated with biodiversity and patterns of yeasts species as reported by Castrillo et al. [62] of grapes from four different areas (Denominations of Origen, DO) of Galicia (Spain). The driest area, DO Monterrey (595 mm), presented lower species richness, dominated by *A. pullulans*, than DO Rias Baixas (1071 mm), dominated by *H. uvarum*. Likewise, the number of yeast species was increased in two Spanish wine zones with both Atlantic and interior climate in a rainy year [63]. Poor sanitary conditions and increased of damaged grapes by cracking could favor the biodiversity of yeasts, especially of oxidative species. In any case, rainy or usual year, yeast biodiversity was greater than our number of yeast species, with 21 and 28 species. In contrast, a reduction of yeast biodiversity was observed with a precipitations increase [61], which could have an effect of washing away yeasts from grapes skin.

Mold populations are frequently dominated by *Alternaria*, *Aspergillus*, *Cladosporium*, *Botrytis,* and *Penicillium* members, although this is also variable depending on the studies. Factors as varieties, climate and cultural practices influence filamentous fungi populations. *Alternaria*, *Botrytis,* and *Cladosporium* were the genera more frequently identified in grapes from regions of Alentejo, Douro, Ribatejo, and Vinhos Verdes in Portugal [21], with climatic conditions similar to those of the present work (Mediterranean climate Csa and Csb according to Köppen climate classification). Serra et al. [21] reported that *Aspergillus* prevalence was dependent on climate, whereas the genus *Penicillium* occurrence was not influenced by climatic conditions. García-Cela et al. [64] showed that *Aspergillus* and *Alternaria* were the dominant genera isolated from Spanish vineyards (Csa according to Köppen climate classification), whereas *Penicillium* isolates represented less than 4% of isolates. In agreement with our results, *Penicillium* was the dominant genus (above 50% of identifications) of molds in grapes from the Burgundy area, with an oceanic climate, in France [4]. Nevertheless, *Penicillium* and *Aspergillus* shared the dominance (50% of identifications) on grapes from 5 regions of France with Oceanic climates (Cfb) such as Alsace, Beaujolais, Côtes du Rhône, Languedoc, and Bordelais [2].

The efforts to control the mold population in vineyards are usually directed to *B. cinerea* [6,36,65], *Alternaria alternaria* [66], mycotoxigenic species of *Aspergillus* section *Nigri* [41,67], and *P. expansum* [44,46]. However, the relevance of other species in the pathogenesis and spoilage of grapes deserves attention [5,47]). In the present work, *Alternaria*, *Cladosporium,* and *Aspergillus* isolates were identified in spoiled grapes, but *P. glabrum* was the most frequent species identified in several bunches with symptoms of *Penicillium* development, where it was the unique species identified. Genetic characterization of *P. glabrum* isolates allowed the identification of two different strains.

In this work, the direct confrontation in plates of PDA among two *P. glabrum* strains and yeasts gave different types of interactions. Inhibitory effects were observed in seven out of 18 isolates of *P. terricola* and one isolate of *A. pullulans* (Figure 2E,F). The strain dependence of biocontrol abilities is in agreement with previous findings of isolates of *I. terricola* (synonymous with *P. terricola*) and *H. uvarum* against *B. cinerea* [60]. Antagonistic activity of strains belonging to *I. terricola* and *A. pullulans* isolated from vineyards has been reported against different pathogens such as *A. carbonarius* and *A. niger* [41,67]; *B. cinerea* [60] and *Greeneria uvicola,* which is responsible for bitter rot [68]. Other isolates such as *A. pullulans* L30 (Figure 2C) and *Z. meyerae* modified the visual appearance of *P. glabrum* (Figure 2D). The other species of yeasts identified did not show remarkable effects on *P. glabrum* development or even those completely covered by mycelia, despite members of *H. uvarum* and *P. kudriavzevii* being previously described as biocontrol agents [52,69]. Moreover, no relevant differences were found in the interactions among yeasts and the two strains of *P. glabrum* evaluated (M1307 and M204). However, in vitro screening of antagonist activity presented disagreement with results obtained in the in vivo assay of wounded grapes (Figure 2 and Figure 3). Yeasts selected as having neutral activity by direct confrontation, *P. kudriavzevii* L18 and *H. uvarum* L35, displayed a remarkable antagonist activity on grapes. Hence, these results suggest that combined strategies must be applied for properly screening antagonist activity.

**Figure 4:**
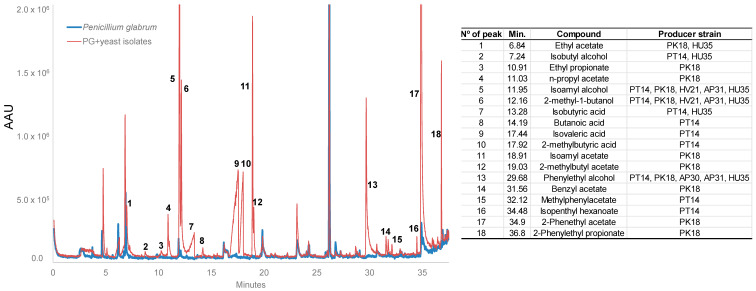
Representative profile of volatile compounds into the double dish systems control (*P. glabrum*, blue line) and confrontations (*P. glabrum* + yeasts; red line). Numbers indicate compounds associated with yeast growth. The table relates the number of peaks, retention times, and identification of compounds (by NIST database, Kovats index) from the producer yeasts.

The inquiry for the possible mechanism of action of selected yeasts showed that the production of antifungal VOCs on PDA plates and its consequent effect on *P. glabrum* growth was a common characteristic in the selected yeasts except for *Z. meyerae* L29, which was without any effect on growth rates, lag phase, and reduction of mycelia diameter. The rest of the yeasts were primarily affected by the lag phase delay, especially to *P. glabrum* M204. Figure 4 shows the volatile compounds associated with yeast development. Acids were found in *P. terricola* L14 confrontations, branched and aromatic alcohols in *H. viticola*, *H. uvarum*, *A. pullulans* and *P. kudriavzevii* confrontations, and esters were associated with *P. kudriavzevii* L18 growth. This last yeast, a producer of branched and aromatic alcohols and their esters, presented the greatest influence on *P. glabrum* growth, which is in agreement with the results obtained by Choińska et al. [69]. Esters such as n-propyl acetate, isoamyl acetate, benzyl acetate, and 2-phenethyl acetate have been associated with *B. cinerea* control [52]. Although *P. kudriavzevii* L18 was previously selected in the direct confrontation as a negative control, this isolate was the most effective antagonist against *P. glabrum* in the in vivo assays. These results pointed out that direct confrontation is not a proper screening method to identify antagonist yeast producers of antifungal VOCs.

Regarding spore germination, the two isolates of the *Pichia* genus reduced the size of the germ tube of *P. glabrum,* and *P. terricola* L14 also inhibited spore germination. This fact could be associated with space colonization, as this was observed in direct confrontation (Figure 2E), whereas reduction of the germ tube size by *P. kudriavzevii* L18 could be associated with antifungal VOC production [70]. The rest of the yeasts did not modify the spore germination.

Concerning the other possible mechanisms of action studied, no parasitism of yeast cells to *P. glabrum* hyphae was observed in most of the selected yeast. A slight attachment of cells of *Z. meyerae* L29 was observed against both strains of molds. Previous studies have proved that the capacity of parasite fungal hyphae is related to the production of lytic enzymes [71]. In this case, chitinase, β-glucanase, and pectinase activity were detected for *Z. meyerae* L29. However, other yeasts with the three hydrolytic capabilities were not related with attachment to fungal hyphae as well as the protease activity shown by *A. pullulans* isolated L30 and L31. Moreover, the results of the in vivo assays reveal that enzymatic activities could not be a key mechanism on fruit surfaces. No antibiosis activities were detected in any yeasts either.

Previous work highlighted the importance of biofilm formation on the biocontrol activity of yeast [27,72], although this remains one of the least known mechanisms of antagonism. Three yeasts, *P. terricola* L14, *Z. meyerae* L29, and *H. uvarum* L35, formed biofilm in vitro assays. This capability has been related to wound colonization in fruits [55]. Our preliminary results showed that biofilm could be involved in the biocontrol of *H. uvarum* L35, which presented remarkable reductions of disease incidence in wounded grapes. However, other mechanisms as the induction of resistance in host tissues [73,74] could be associated with a reduction of disease development by *H. uvarum* L35.

The competition for nutrients is another relevant mechanism of biological control. In our study, iron presence did modify the antagonism action on selected yeast. The enrichment of grape wounds with YNB increased the incidence of *P. glabrum* (from ~10% infections to ~90% of inoculated grapes) in confrontations with *P. terricola* L14, proving that this yeast bases its antagonism on the competition for nutrients more than biofilm formation. Using the same methodology, Ruiz-Moyano et al. [53] showed that *Metschnikowia pulcherrima* L672 based its mechanism on the competition for nutrients.

Finally, characterization of the mechanisms of antagonism explained, at least partially, the results obtained in the inoculation of wounded grapes, where five yeasts efficiently reduced the infection of *P. glabrum* (Figure 3). Other mechanisms may be involved; so, further research is needed to clarify them.

## 5. Conclusions

During development in vineyards and subsequent processes and commercialization, grapes are infected by different pathogenic molds. In this work, *P. glabrum* was the dominant mold in spoiled bunches.

To control the infections and spoilage by *P. glabrum,* a screening of potential antagonistic yeasts was performed. Five yeasts, *P. terricola* L14, *Z. meyerae* L29, *P. kudriavzevii* L18, *H. viticola* L21, and *H. uvarum* L35, efficiently reduced the infection of *P. glabrum* on wounded grapes, although *P. terricola*, *H. viticola* and *H. uvarum* themselves triggered different symptoms of alteration on grapes. Different mechanisms, such as the production of VOCs, secretion of lytic enzymes, biofilm formation, and competition for nutrients, were involved in biocontrol. Finally, the isolated *P. kudriavzevii* L18 (a producer of antifungal VOCs) and *Z. meyerae* L29 (with chitinase, β-glucanase and pectinase activities and a producer of in vitro biofilm) are proposed as biocontrol agents in vineyards and during the commercialization of grapes. To our knowledge, this is the first report about the potential of *Z. meyerae* as a biocontrol agent.

## Figures and Tables

**Table 1:** Yeast species identification (species, percentage of identity, and accession number of reference), number of isolates, and presence of the species per 10 bunches.

N° Isolates	Presence Per Ten Bunches	Species Identification	Percentage of Identity	Accession Number
Yeasts				
13	10	*Pichia terricola*	100	NR153994
8	6	*Pichia kudriavzevii*	100	KY104583
17	10	*Aureobasidium pullulans*	100	KX869960
8	4	*Hanseniaspora uvarum*	99.86	KY103573
6	2	*Hormonema viticola*	99.82	NR137620
2	1	*Zygoascus meyerae*	100	KY106012
Filamentous fungi				
18	10	*Penicillium glabrum*	100	AY373915
1	1	*Cladosporium cladosporioides*	100	HQ832794
11	4	*Alternaria alternata*	100	MG733652
3	2	*Aspergillus nigricans*	100	KP124341

**Table 2:** Growth parameters (μ and lag phase) and percent reduction of mycelia of *P. glabrum* confronted with selected yeasts for the detection of antifungal of volatile organic compounds (VOCs).

Confrontation	(mm Day^−1^)		Lag Phase (Day^−1^)		% Reduction in Size of Mycelia (Day 4)	
	Media	SD ^1^	Media	SD	Media	SD
*Penicillium glabrum* 1307						
Control	5.45 ^d,e,f,1^	0.07	0.86 ^e,f^	0.01	-	-
*Pichia terricola* L14	5.77 ^a,b,c,d^	0.29	1.52 ^a,b^	0.19	9.17 ^b^	4.00
*Pichia kudriavzevii* L18	4.73 ^g^	0.25	1.65 ^a^	0.12	31.48 ^a^	2.50
*Hormonema viticola* L21	4.94 ^f,g^	0.07	0.87 ^e,f^	0.03	6.06 ^b,c,d^	3.61
*Zygoascus meyerae* L29	5.77 ^a,b,c,d^	0.14	1.11 ^c,d,e^	0.09	−0.10 ^d,e^	5.73
*Aureobasidium pullulans* L30	5.72 ^b,c,d^	0.16	1.03 ^d,e^	0.04	−1.65 ^e^	3.75
*Aureobasidium pullulans* L31	5.59 ^c,d,e^	0.09	1.00 ^d,e^	0.08	−2.38 ^e^	2.60
*Hanseniaspora uvarum* L35	5.51 ^d,e^	0.08	1.02 ^d,e^	0.09	1.38 ^c,d,e^	6.02
*Penicillium glabrum* 204						
Control	5.51 ^e,f^	0.16	0.61 ^e^	0.09	-	-
*Pichia terricola* L14	5.30 ^e,f,g^	0.04	1.02 ^d,e^	0.03	9.842 ^b^	3.34
*Pichia kudriavzevii* L18	5.10 ^f,g,h^	0.24	1.35 ^a,b^	0.11	23.91 ^a^	1.68
*Hormonema viticola* L21	6.18 ^a,b,c^	0.05	1.22 ^b,c^	0.04	11.23 ^b^	1.96
*Zygoascus meyerae* L29	5.85 ^d,e^	0.14	0.77 ^e^	0.08	2.69 ^c,d,e^	3.05
*Aureobasidium pullulans* L30	6.27 ^a,b^	0.26	1.24 ^a,b,c^	0.11	2.08 ^c,d,e^	4.44
*Aureobasidium pullulans* L31	6.08 ^a,b,c,d^	0.31	1.08 ^b,c,d,e^	0.21	7.00 ^b,c^	2.05
*Hanseniaspora uvarum* L35	6.41 ^a^	0.04	1.33 ^a,b^	0.02	7.37 ^b,c^	1.95
*p*	<0.001		<0.001		<0.001	

^1^ statistical deviation. ^a–h^ by columns, media values with different letters indicates significant differences (*p* < 0.050).

**Table 3:** Percentage of germinated spores and size of germ tube (μM) in direct contact with selected yeasts.

Confrontation		% Spores Germinated						Size of Germ Tube (μM)					
		10^5^ Cell mL^−1^		10^6^ Cell mL^−1^		10^7^ Cell mL^−1^		10^5^ Cell mL^−1^		10^6^ Cell mL^−1^		10^7^ Cell mL^−1^	
		Media	SD ^1^	Media	SD	Media	SD	Media	SD	Media	SD	Media	SD
*Penicillium glabrum* 1307													
	Control	88.78			4.03			71.61			30.50		
	*Pichia terricola* L14	77.45 *	5.32	65.97 *	8.68	64.88 *	5.25	35.13 *	12.42	26.06 *	9.088	27.93 *	7.16
	*Pichia kudriavzevii* L18	77.31	10.60	81.72	3.91	81.04	6.46	38.26 *	11.31	34.20 *	17.61	35.13 *	14.70
	*Hormonema viticola* L21	91.28	6.58	93.44	5.10	92.52	5.01	74.23	32.42	85.73	37.72	68.46	37.55
	*Zygoascus meyerae* L29	93.22	4.36	93.87	4.10	94.36	3.75	77.13	28.73	73.53	36.32	73.82	36.33
	*Aureobasidium pullulans* L30	90.93	3.89	92.49	4.90	91.56	5.78	84.20	38.48	73.46	33.67	80.27	37.05
	*Aureobasidium pullulans* L31	93.61	1.83	93.59	4.23	87.53	3.92	67.60	24.58	66.26	15.10	61.66	22.46
	*Hanseniaspora uvarum* L35	94.13	0.61	91.48	4.88	92.18	5.53	72.40	28.96	77.49	27.99	76.46	18.67
*Penicillium glabrum* 204													
	Control	93.74			3.93			87.02			42.20		
	*Pichia terricola* L14	75.50 *	3.42	70.84 *	2.82	63.61 *	7.28	36.46 *	12.40	29.46 *	17.53	29.13 *	15.45
	*Pichia kudriavzevii* L18	87.02	2.68	88.25	3.16	90.54	6.12	48.40 *	20.20	42.40 *	24.23	37.33 *	18.18
	*Hormonema viticola* L21	93.44	4.87	90.68	3.24	89.65	3.84	76.46	24.73	84.02	30.53	62.93	29.32
	*Zygoascus meyerae* L29	90.84	4.46	89.56	5.91	89.69	3.84	63.66	27.15	66.06	22.90	66.61	17.12
	*Aureobasidium pullulans* L30	86.71	5.87	90.67	4.70	88.58	4.75	101.2	15.86	72.26	26.35	64.13	20.53
	*Aureobasidium pullulans* L31	92.74	7.21	88.28	4.58	84.35	10.50	65.66	25.76	69.01	28.62	80.66	24.07
	*Hanseniaspora uvarum* L35	97.89	2.88	95.69	4.23	92.08	5.96	71.13	29.59	62.42	22.52	64.06	15.91
	*p*	<0.001		<0.001		<0.001		<0.001		<0.001		<0.001	

^1^ statistical deviation. * statistical differences with control (*p* < 0.050).

**Table 4:** Assays for characterization of biocontrol mechanism.

Strain.	Parasitism	Antibiosis	Enzymatic Activities				Biofilm Formation	Competition for Nutrients	
			Chitinase	β-Glucanase	Pectinase	Protease		Iron Depletion	In Vivo Competition
*Pichia terricola* L14	−	−	−	+	−	−	+	−	+
*Pichia kudriavzevii* L18	−	−	−	−	−	−	−	−	−
*Hormonema viticola* L21	−	−	+	+	+	−	−	−	−
*Zygoascus meyerae* L29	−	−	+	+	+	−	+	−	−
*Aureobasidium pullulans* L30	−	−	+	+	+	+	−	−	−
*Aureobasidium pullulans* L31	−	−	+	+	+	+	−	−	−
*Hanseniaspora uvarum* L35	−	−	−	−	−	−	+	−	−
